# Social Connectedness Across the Psychosis Spectrum: Current Issues and Future Directions for Interventions in Loneliness

**DOI:** 10.3389/fpsyt.2014.00154

**Published:** 2014-11-11

**Authors:** Michelle H. Lim, John F. Gleeson

**Affiliations:** ^1^Brain and Psychological Sciences Centre, Swinburne University of Technology, Hawthorn, VIC, Australia; ^2^Australian Catholic University, Melbourne, VIC, Australia

**Keywords:** loneliness, psychosis continuum, social relationships, cognition, treatment

Loneliness, sometimes referred to as “perceived social isolation,” is defined as a subjective experience of social isolation. Loneliness has been shown to be related more to the quality of social relationships than to the quantity, and is typically characterized by feelings of social disconnection (e.g., being misunderstood by others). It occurs when there is a discrepancy between desired and actual amounts of social interaction. Humans are a social species and have a fundamental need to belong. Feelings of loneliness have been perceived to be early warning signals of potential threats to psychological health (akin to physical pain in physical health problems). Loneliness is associated with an increased risk of various health conditions (e.g., increased inflammation, decreased immunity) and can occur in transient and chronic forms across the lifespan.

In the last few decades, there has been an increase in scientific studies in loneliness and much of this research stemmed from a social neuroscience approach (see work by Cacioppo). The onset of loneliness is thought to motivate an individual to seek connectedness with others; however, symptoms of mental illness often involve withdrawal from the social world. A growing interest in the relationship between loneliness and mental health disorders is therefore not surprising and was first identified as an important relationship in the late 1950s. Psychoanalyst Frieda Fromm-Reichmann highlighted the devastating impact of loneliness on patients with schizophrenia. In more recent times, researchers have used a neuroscience approach to further clarify the relationship between social withdrawal/isolation and positive symptoms of psychosis (see Hoffman’s social deafferentation hypothesis).

The psychological consequences of loneliness, however, remain under examined by researchers. It is plausible, but remains unclear, that loneliness is a transdiagnostic factor across different mental disorders that raises the risk of mental health problems, increases the severity of symptoms, maintains diagnostic status, or all of the above. Loneliness is associated with various mental disorders, including depression, social anxiety disorder, and obsessive–compulsive disorder (1), and most of the research on loneliness and mental health has focused on its relationship with depression (2).

To date, there has been no published study that has developed an evidence-based loneliness intervention in individuals with psychosis. A meta-analytic review of interventions aimed at reducing loneliness in a range of different populations surprisingly included only five studies with individuals with mental health symptoms, and none of the studies were specific to psychosis (3). There is, however, emerging research that highlights the deleterious effects of loneliness in individuals with psychotic disorders. In the second Australian national survey of psychosis (*N* = 1825), 80.1% of adults aged between 18 and 34 years, diagnosed with a psychotic disorder endorsed perceived loneliness; 37.2% of these adults identified loneliness as a barrier to recovery (4). While many psychosocial interventions are aimed at introducing new social supports (e.g., befriending) or providing social skills training (SST) for people with psychotic disorders, there has been no known study that specifically targets loneliness.

## Loneliness and the Psychosis Spectrum

Psychotic symptoms occur on a continuum, ranging from the absence of symptoms to the sustained presence of clinically distressing symptoms. The term “delusion-prone individuals” refers to individuals who report delusional ideation but who are not clinically delusional because of a lack of functional impairment or distress associated with their beliefs. One identified delusion-prone group consists of members of particular new religious movements (NRMs). We found that, despite reporting similar levels of delusional ideation to individuals with psychosis, individuals in the NRM group were not as distressed by their beliefs. One factor that may explain this attenuated distress is the nature of the social relationships under study. NRM individuals reported significantly more helpful supports and more crisis supports than individuals with psychosis. Having a strong group identity may be a protective factor against distress (5). Acceptance into a group of individuals who hold similar values is likely to generate feelings of connectedness and increase the chance of having a confidant from whom one can seek support.

The specific dynamics of relationships (e.g., reciprocity) held can further moderate distress associated with delusional ideation. Reciprocity refers to an exchange–style relationship between two individuals in which both individuals seek support from each other. In our study, participants with psychosis who reported higher relationship reciprocity were significantly less distressed than those who reported lower relationship reciprocity. One possible explanation is that more balanced relationships may promote positive bonds between individuals whereas less balanced (or one-sided) relationships may confer feelings of burden on the helper and guilt on the recipient. To facilitate the development of more balanced relationships, individuals with psychosis may benefit not just from receiving social support but also from opportunities to in turn provide constructive social support in ways that improve their self-esteem.

Connecting with peers and establishing reciprocal relationships in a naturalistic environment (i.e., less structured social settings that can engender hope and spontaneity) are crucial to buffer against distress associated with psychosis. However, there are potential barriers that should be considered, such as co-occurring social anhedonia, social withdrawal, and schizotypy traits associated with psychotic disorders. In our study, participants with psychosis did not report significantly more dissatisfaction with their relationships, despite reporting significantly fewer and less helpful relationships in their network when compared to the delusion-prone group. It is possible that individuals with psychosis may: (a) be unable to identify a need to initiate or maintain friendships due to negative symptoms or maladaptive cognitions about the social world; or (b) be too distressed to meaningfully participate in social interventions due to active symptoms compared to those in remission or those who report subthreshold psychotic symptoms.

## Future Directions in Interventions for Loneliness

The early stages of a psychotic disorder can be an isolating time for those afflicted and often entails debilitating social consequences. Targeting loneliness is likely to alleviate distress associated with psychotic symptoms. A well-designed intervention is warranted and may be informed by the following guidelines.

### Addressing maladaptive cognition in social relationships

The experience of loneliness has been found to be “socially contagious” within social networks; in other words, lonely individuals are connected to other lonely individuals (6). Connecting lonely individuals with other lonely individuals may not necessarily lead to them creating friendships because they demonstrate thoughts and behaviors that are unconducive to friendship development. This highlights the importance of addressing maladaptive cognitions arising from the ineffective navigation of the social world.

Because of its subjective nature, loneliness is driven (or at least maintained) by biased cognitions related to the social world, including negative interpretations of social interactions and beliefs about others. In a cognitive model of loneliness, maladaptive cognitions are influenced by other processes, including hypervigilance to social threats and various cognitive biases [e.g., memory bias, confirmatory biases (7)]. In brief, lonely individuals are more likely to form more negative impressions and show more punitive behaviors toward others. Lonely individuals actively contribute to the vicious cycle of loneliness through their use of self-protective behaviors and having self-defeating interactions with others, further isolating themselves.

Addressing maladaptive cognitions around pre-existing social networks may be a useful starting point to improving the quality of those relationships. Any social skills deficits that inhibit the quality of current relationships can also be easily identified and quickly addressed. It may be more feasible for those with high avoidance tendencies (e.g., comorbid social anxiety or schizotypy) to improve current relationships rather than develop new relationships.

Another advantage of looking within current social networks is to identify opportunities to develop a confidant relationship with a known individual or to improve the relationship with a current confidant. The absence of confidants has been linked to higher loneliness in a community sample (8). Individuals with first-episode psychosis were less likely to have a confidant than healthy controls (9). Individuals with psychosis tend to confide in a family member (over 40%) as opposed to a friend (over 30%); others reported that they lost their confidant in the previous 12 months (12.7%) or never had one (15.6%). Almost half (over 48%) of those surveyed reported that they needed more friends (4). Developing and/or improving a relationship with a confidant within the existing social network may be a stepping-stone to developing connections with unfamiliar individuals.

### Using positive affect to enhance social bonds

Lonely individuals, when compared to less lonely individuals, may have the necessary social skills to relate to others but may not readily use or recognize the efficacy of those skills. However, for people with psychosis (a disorder associated with social skills deficits), it may be crucial to incorporate specific components of SST for loneliness to be successfully targeted. For example, SST components that focus on developing positive interpersonal styles may help people with psychosis to establish more stable social bonds with others.

To date, there has been minimal research on the effectiveness of positive interpersonal styles (e.g., sharing successes in day-to-day life) for mental well-being. There is growing interest in positive psychology principles, specifically in the nature of positive affect in individuals with mental disorders. Positive affect appears to be attenuated in individuals with psychosis. Specifically, these individuals were less likely to savor past or future positive experiences, possibly contributing a lack of engagement with others (10). More severe levels of negative symptoms in individuals with psychosis are associated with having fewer friendships (11). A limited ability to savor positive experiences and the presence of negative symptoms should be accounted for when developing a social intervention for individuals with psychosis. One suggestion to combat the effects of negative symptoms is to teach savoring techniques and to improve self-efficacy pertaining to interpersonal behavior skills.

Another way to generate positive affect is to practise interpersonal styles that can be used to enhance social bonds. The concept of capitalization is borrowed from interpersonal styles in romantic relationships. Capitalization is defined as the ability to seek out others when positive things occur (12). The ability to capitalize and provide constructive responses to positive events may cultivate positive affect and enhance the bonds between two people within a relationship. For example, in romantic relationships, capitalization is associated with higher relationship well-being (e.g., intimacy). The ability to self-disclose is another factor that may have been under examined in current social interventions. Emerging research has indicated that self-disclosure is integral to relationship development; specifically, disclosure reciprocity that is greater and more immediate than not is associated with more liking and closeness in relationships (13).

### Increase accessibility to a positive social environment

Individuals who are connected with lonelier individuals also become lonelier themselves over time, demonstrating the powerful influence of social networks. Lonely individuals embedded in an enriched social environment find it easier to break out of the loneliness cycle than those without such an enriched environment. Unfortunately, individuals with psychosis are well-known to have impoverished social networks, and the ability to connect with others may be further limited by additional environmental factors such as societal stigma. While access to a social environment where one can practise positive social interactions and form strong social bonds with others is more complex for individuals with psychosis, it is not unachievable and will likely mitigate loneliness once established. Researchers can also consider the use of technology, such as moderated online social forums, to reach individuals who find it difficult to participate in a new social environment. Although online communication forms have become the norm are advantageous in terms of accessibility and can be used as a transitional medium toward in-person communication, the caveats to online communication should be noted (e.g., forums should be moderated to facilitate a positive and safe social environment). Regardless of the modality, it is crucial that people with psychosis be given easy access to a naturalistic social environment that engenders hope and spontaneity, and provides a platform where positive relationships can be nurtured (i.e., practise capitalization, and so on).

## Conclusion

In sum, loneliness hurts. The aversive experience of loneliness, together with well-known physical and mental health risks, justify the development of specific interventions targeting the reduction of loneliness. Unfortunately, the crucial relationship between loneliness and psychosis has been overlooked and under examined. Individuals with psychosis often suffer myriad difficulties that may fuel loneliness. The ability to connect with others is challenged by various factors ranging from the nature of the psychosis presentation (e.g., negative symptoms) to environmental factors (e.g., societal stigma). There is a crucial need to design an empirically sound intervention for loneliness for individuals with psychosis. It is plausible that a well-designed intervention may reduce the risk of developing psychosis, alleviate the distressing experience of acute psychotic symptoms, and reduce the risk of relapse of psychotic symptoms.

## Conflict of Interest Statement

The authors declare that the research was conducted in the absence of any commercial or financial relationships that could be construed as a potential conflict of interest.
